# Improvement of prognostic performance in severely injured patients by integrated clinico-transcriptomics: a translational approach

**DOI:** 10.1186/s13054-015-1127-y

**Published:** 2015-11-26

**Authors:** Daniel Rittirsch, Veit Schoenborn, Sandro Lindig, Elisabeth Wanner, Kai Sprengel, Sebastian Günkel, Barbara Schaarschmidt, Sonja Märsmann, Hans-Peter Simmen, Paolo Cinelli, Michael Bauer, Ralf A. Claus, Guido A. Wanner

**Affiliations:** Division of Trauma Surgery, Department of Surgery, University Hospital Zurich, University of Zurich, Raemistrasse 100, CH-8091 Zurich, Switzerland; Department of Anaesthesiology and Intensive Care Therapy, Jena University Hospital, Erlanger Allee 101, D-07747 Jena, Germany; Center for Sepsis Control and Care, Jena University Hospital, Erlanger Allee 101, D-07747 Jena, Germany

## Abstract

**Introduction:**

Severe trauma triggers a systemic inflammatory response that contributes to secondary complications, such as nosocomial infections, sepsis or multi-organ failure. The present study was aimed to identify markers predicting complications and an adverse outcome of severely injured patients by an integrated clinico-transcriptomic approach.

**Methods:**

In a prospective study, RNA samples from circulating leukocytes from severely injured patients (injury severity score ≥ 17 points; n = 104) admitted to a Level I Trauma Center were analyzed for dynamic changes in gene expression over a period of 21 days by quantitative RT-PCR. Transcriptomic candidates were selected based on whole genome screening of a representative discovery set (n = 10 patients) or known mechanisms of the immune response, including mediators of inflammation (IL-8, IL-10, TNF-α, MIF, C5, CD59, SPHK1), danger signaling (HMGB1, TLR2, CD14, IL-33, IL-1RL1), and components of the heme degradation pathway (HP, CD163, HMOX1, BLVRA, BLVRB). Clinical markers comprised standard physiological and laboratory parameters and scoring systems routinely determined in trauma patients.

**Results:**

Leukocytes, thrombocytes and the expression of sphingosine kinase-1 (SPHK1), complement C5, and haptoglobin (HP) have been identified as markers with the best performance. Leukocytes showed a biphasic course with peaks on day 0 and day 11 after trauma, and patients with sepsis exhibited significantly higher leukocyte levels. Thrombocyte numbers showed a typical profile with initial thrombopenia and robust thrombocytosis in week 3 after trauma, ranging 2- to 3-fold above the upper normal value. ‘Relative thrombocytopenia’ was associated with multi-organ dysfunction, the development of sepsis, and mortality, the latter of which could be predicted within 3 days prior to the time point of death. SPHK1 expression at the day of admission indicated mortality with excellent performance. C5-expression on day 1 after trauma correlated with an increased risk for the development of nosocomial infections during the later course, while HP was found to be a marker for the development of sepsis.

**Conclusions:**

The combination of clinical and transcriptomic markers improves the prognostic performance and may represent a useful tool for individual risk stratification in trauma patients.

**Electronic supplementary material:**

The online version of this article (doi:10.1186/s13054-015-1127-y) contains supplementary material, which is available to authorized users.

## Introduction

The acute inflammatory response is organized within a highly complex “network of inflammation” [1], which is carefully orchestrated under regular conditions and is required for post-injury regeneration and tissue repair. However, in the case of an overwhelming initial insult loss or failure of control, mechanisms can lead to systemic inflammation with additional harm to host cells and organs, eventually resulting in multiorgan failure (MOF) [2, 3]. Based on previous research, different models for the inflammatory response following major trauma have been conceptualized, all of which have in common that the underlying pathophysiology and molecular mechanisms of the host response are responsible for adverse events and a complicated recovery [4–8]. The pathophysiology of systemic inflammation is thus taken into account in contemporary treatment concepts, such as damage control surgery [9–12]. However, specific immune modulatory therapies for the treatment of severely injured trauma patients and septic patients could not be established to date. Furthermore, clinical decision-making is still based on general, unspecific physiologic parameters and the physicians’ experience. Despite extensive research in the past, only C-reactive protein (CRP), procalcitonin (PCT), and—to a lesser extent—interleukin (IL)-6 found their way into routine clinical use for assessment of the immune response in trauma-induced systemic inflammation and sepsis [12, 13].

Initial research focused on detection of circulating mediators of inflammation that are released upon severe trauma, and the initial inflammatory response was commonly referred to as a “cytokine storm” [14]. Meanwhile, it has become evident that the host response comprises complex interactions between inflammatory, humoral, neurological, and endocrine systems [1, 9]. This is reflected by novel approaches for a better understanding of the pathophysiology, including large-scale genomic, proteomic, and cellular immune signatures [5, 15, 16]. In contrast to previous studies which focused on the role of individual mediators and mechanisms, new research directions aim for a systemic perspective at the proteomic level as well as the genomic level. These recent studies revealed the complexity of the transcriptomic events underlying inflammation, but the applicability of this information in the clinical setting is still limited. We postulated that by combining gene expression changes with routinely used clinical and laboratory parameters it would be possible to improve the prognostic performance.

On the one hand, candidate genes were selected based on previous knowledge of their role in the pathophysiology in systemic inflammation, including danger-associated molecular patterns (high mobility group box protein-1 (HMGB1) [17, 18], IL-33 [19, 20]), interleukin-1 receptor-like 1 (IL-1RL1, ST2) [19, 20], components of the complement system (C5) [21, 22], sphingosine kinase (SPHK)-1 [23, 24], and selected cytokines (tumor necrosis factor alpha (TNFα) [25, 26], macrophage migration inhibitory factor (MIF) [27–29], IL-8 [30], IL-10 [31]). On the other hand, candidate selection was based on whole genome analyses of a representative discovery set, which comprised genes of the pathogen-recognition receptors (toll-like receptor (TLR) 2); CD14) and the complement system (CD59), as well as members of the heme degradation pathway (haptoglobin (HP), CD163, heme oxygenase-1 (HMOX1), biliverdin reductase (BLVR) A and B). With this approach we could show that the combination of clinical and transcriptomic markers (clinico-transcriptomic analyses) improves the prognostic performance and may represent a useful tool for individual risk stratification in trauma patients.

## Materials and methods

### Study design

**Fig. 1:**
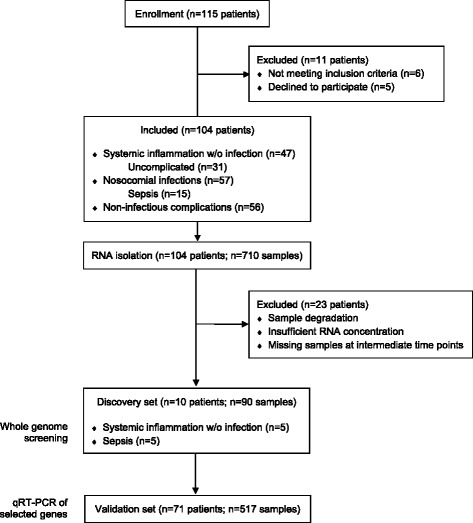
CONSORT flow diagram of the study design. *qRT-PCR* quantitative RT-PCR, *w/o* without

Blood was sampled from 104 patients with multisystem trauma admitted to the Division of Trauma Surgery (level I trauma center) at the University Hospital Zurich from December 2009 to March 2012. Criteria for study enrollment included patient age ≥18 years, an Injury Severity Score (ISS) ≥17 points, and time from injury to admission <6 hours. All patients were recruited into the study under informed consent guidelines approved by the Cantonal Ethic Commission Zurich (StV 26-2007) and international ethical guidelines (ClinicalTrials.gov: NCT02508272). Study subjects were treated under the guidance of standard operating procedures developed and implemented at the University Hospital Zurich (based on guidelines of the German Society of Trauma (DGU) [32]). Whole blood from trauma patients was collected within the first 6 hours after trauma (day 0) and on days 1, 2, 3, 5, 7, 10, 14, and 21. Clinical outcomes and complications within 28 days after injury were recorded. To illustrate the underlying study design, a CONSORT flow diagram is displayed in Fig. 1. The total cohort consists of 104 trauma patients. For analysis of clinical and laboratory parameters, all 104 patients were included. Ten of 104 patients, with unambiguous clinical presentation with respect to the development of sepsis or systemic inflammation without infection, were selected as a representative discovery set (*n* = 10 patients; *n* = 90 samples) which was analyzed by whole genome screening in a recent study [GEO:GSE70311]. Candidate genes were identified by standard statistical methods for analysis of microarray datasets: gene set enrichment analysis (GSEA) was performed using hypergeometric tests with FDR correction within the GeneAnswers, https://www.bioconductor.org/ package mapped to Reactome pathways and Gene Ontology (GO) categories. Further statistical procedures comprised explorative gene set analysis and principle component analysis. After candidate gene selection and exclusion of patients with degraded or missing samples at intermediate time points, candidate genes were validated in the total cohort (*n* = 71 patients; *n* = 517 samples) by quantitative RT-PCR. The rates of adverse outcomes for either group are indicated in Table 1.

**Table 1:** Patient characteristics

Parameter	Total cohort (*n* = 104)	PCR cohort (*n* = 71)
Demographics		
Age (years)	43.5 ± 1.77; 42 (18–92)	41.6 ± 2.0; 40 (18–80)
Sex (male/female)	77/27	52/19
Glasgow Coma Scale	11.9 ± 0.41; 14 (3–15)	11.9 ± 0.5; 14 (3–5)
Injury Severity Score	32.8 ± 1.3; 31 (17–75)	31 ± 1.5; 29 (13–75)
SOFA score initial	4.8 ± 0.3; 5 (0–12)	4.19 ± 0.4; 4 (0–12)
SOFA score maximum	7.7 ± 0.4; 8 (0–18)	6.9 ± 0.5; 7 (0–18)
Outcomes		
RISC (% survival)	83.3 ± 2.5	85 ± 2.8; 96.3 (9.8–98.9)
Survival	88 % (13 nonsurvivors)	90 % (7 nonsurvivors)
Hospital length of stay (days)	26.6 ± 1.8; 21.5 (2–119)	25 ± 2.1; 20 (3–119)
Intensive care unit length of stay (days)	13.9 ± 1.4; 10 (2–86)	11.2 ± 1.2; 8 (2–47)
Allogenic blood transfusion		
TASH score (points)	8.5 ± 0.6; 8 (0–23)	7.8 ± 0.7; 7 (0–23)
TASH (%)	13.9 ± 2.0	12.4 ± 2.2; 5 (5–82)
Initial (day 0) pRBC transfusion (units)	4.0 ± 0.8; 1 (0–54)	3.1 ± 0.7; 0 (0–28)
Total pRBC transfusion	9.6 ± 1.3; 5 (0–70)	7.8 ± 1.2; 4 (0–58)
Infectious complications		
Nosocomial infections	56/104 (53.9 %)	34/71 (48 %)
Sepsis	15/104 (14.0 %)	10/71 (14.1 %)

Data presented as mean ± standard error of the mean; median (minimum–maximum)

*SOFA* Sequential Organ Failure Assessment, *pRBC* Packed Red Blood Cells, *RISC* Revised Injury Severity Classification Score, *TASH score* Trauma Associated Severe Hemorrhage Score

### Clinical data

Clinical data were collected daily in a prospective manner. The occurrence and severity of systemic inflammation, sepsis, MOF, and nosocomial infections were retrospectively analyzed using the corresponding clinical parameters and scores from patients’ records. Systemic inflammation was defined according to criteria of the American College of Chest Physicians/Society of Critical Care Medicine Consensus Conference [2, 33]. For assessment of the severity of trauma-induced systemic inflammation a scoring system was used (Additional file 1: Table S1) [34]. Based on this Systemic Inflammation score (SI score), secondary sepsis in trauma patients was defined as ∆SI score (difference of SI score between two consecutive time points) ≥ +2 points in concomitance with an infectious focus or positive blood cultures. MOF was defined according to the Sequential Organ Failure Assessment (SOFA) score [35].

### RNA isolation

PaxGene (PreAnalytix, Hombrechtikon, Switzerland) tubes were used for sampling and preservation of whole blood, and total cellular RNA from circulating leukocytes was isolated (PaxGene Blood RNA Kit; PreAnalytix) in a Qiacube apparatus (Qiacube, Hilden, Germany) according to the manufacturer’s instructions. RNA integrity was proven using Experion (Biorad, Munich, Germany) microcapillary electrophoresis. Samples exhibiting a RNA quality indicator number (RQI) >7.5 (calculated by Experion System Operation and Data Analysis Tool; Biorad) were included and processed. Most of the isolated totRNA samples met these requirements, with the exception of four out of 710 samples (four patients); more than 90 % had RQI >8.5. Some of the samples contained insufficient RNA concentrations for reverse transcription. These patients were therefore excluded from the study (31 samples, *n* = 16 different patients). An additional three patients were excluded because of missing samples at early or intermediate time points. In total, 23 patients were excluded from the analyses. For cDNA synthesis, 1 μg total RNA/sample were transcribed (RevertAid First Strand cDNA Synthesis; ThermoFisher Scientific, Schwerte, Germany; and PTC-200 Thermal Cycler Dual; BioRad) according to the manufacturer’s protocol. Only patients with missing or degraded total RNA samples at intermediate time points (before the end of the observation period, or prior to discharge or death of the patient), resulting in discontinuous sampling, were excluded. After quality control and exclusion of degraded samples, patients completely unimpaired RNA sample sets at all time points (*n* = 71 patients) were subjected to quantitative RT-PCR.

### Quantitative RT-PCR

Quantitative RT-PCR was performed in a two-step protocol using the Rotor-Gene system and Rotor-Gene SYBR Green PCR Kit (Qiagen, Hombrechtikon, Switzerland) according to the manufacturer’s information with 250 nM Primer mix and 25 ng cDNA. Initial denaturation was at 95 °C for 5 minutes, followed by 50 cycles of denaturation at 95 °C for 5 seconds and annealing/extension at a given temperature (see Additional file 2: Table S2) for 15 seconds, finally followed by a melting curve. Cycle threshold (CT) values and efficiency were documented for each sample, and data were normalized using the housekeeping gene *ACTB*:

$$ \Delta \mathrm{C}\mathrm{T} = \mathrm{C}\mathrm{T}\left[ ACTB\right]\hbox{--} \mathrm{C}\mathrm{T}\left[\mathrm{Candidate}\right]. $$

Primers were purchased from Biomers (Ulm, Germany). The primer sequences are listed in Additional file 2: Table S2.

### Statistical analysis

Comparisons of ∆CT values for the various groups were displayed in box-whisker plots. Significance was attained at *p* <0.05 using the Mann–Whitney U (Wilcoxon rank sum) test. Similarity was assessed using parametric (*r*) and nonparametric (ρ) measures. Differences in time courses were assessed by two-way analysis of variance (ANOVA). A bivariate greedy search algorithm was applied for identification and ranking of the best candidates regarding their performance, which was further characterized by receiver operating characteristic (ROC) curve analysis. Analyses were performed using R software version 3.1.1 (http://www.r-project.org/) and GraphPad Prism 5 (GraphPad Software Inc., La Jolla, CA, USA). Multivariate analyses, including ANOVA, multivariate linear models with post hoc-corrected *p* values, and lagged correlation analyses of various clinical parameters (leukocytes, platelets, sepsis, SI score, time, mortality, gender, age, etc.) and candidate gene expression, have been employed. For cluster analysis Fig. 6, time index of peak measurements were used in order to evaluate and illustrate common features and expression patterns and their temporal relationships in patients with a similar clinical course and outcome with respect to nosocomial infections and sepsis. Machine learning was applied for decision tree generation by 10-fold cross-validation. Decision trees/candidates were selected upon high specificity.

## Results

### Patient population

Characteristics of the patient cohort are presented in Table 1. A total of 104 trauma patients with an ISS ≥17 points were enrolled in the study. The mean ISS was 32.8 points. The leading injury mechanism was blunt trauma. Thirteen of 104 patients died within the observation period of 28 days (mortality rate 12 %). Sepsis occurred in 15 of 104 patients (14 %). Fifty-six patients developed nosocomial infections during hospitalization (54 %), including ventilator-associated pneumonia, surgical site infections, and urinary tract infections as the most frequent causes (for time points of sepsis diagnosis and death (see Additional file 3: Table S3). 

### Leukocytes reflect the severity of systemic inflammation and correlate with the development of sepsis, while thrombocytes are associated with an adverse outcome in general

**Fig. 2:**
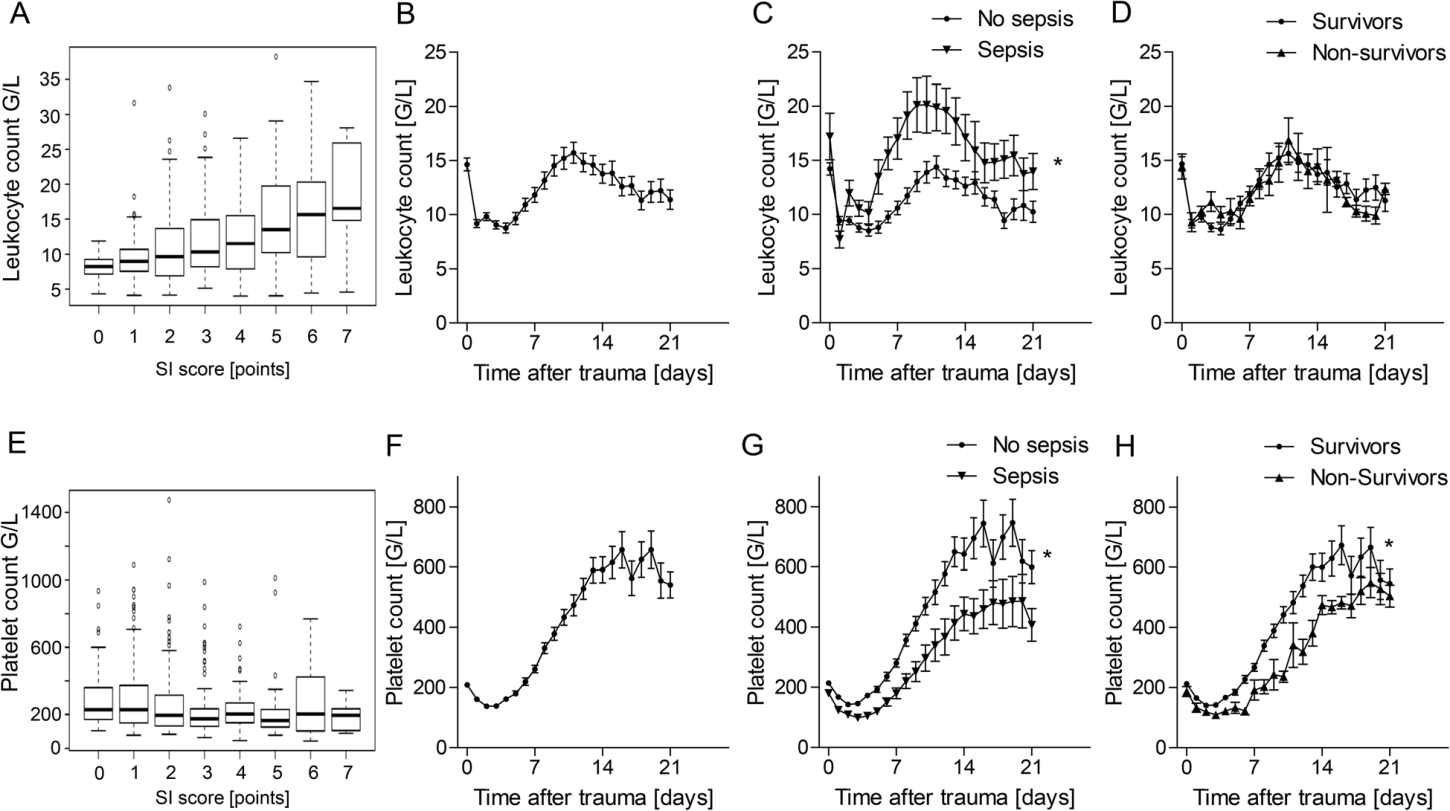
Systematic analysis of leukocyte **a–d** and thrombocyte counts **e–h** in trauma patients (*n* = 104 patients). **a, e** Correlation with the severity of systemic inflammation (*SI* score). **b, f** Time course of the total cohort. **c, g** Subgroup analysis of patients with or without sepsis as a function of time. **d, h** Comparison of time courses of survivors and nonsurvivors. *p <0.05

After severe trauma, leukocyte and thrombocyte counts underlie a dynamic regulation that starts immediately after the initial injury and is affected by multiple conditions, such as consumption during hemorrhagic shock and coagulopathy, bone marrow activation, or induction of processes necessary for tissue regeneration and repair. While the predictive value of leukocyte levels and thrombocytopenia is well established in sepsis in nontrauma patients, to our knowledge a systemic longitudinal analysis in trauma is not available. We therefore first correlated the changes in leukocyte counts during the course of time. As displayed in Fig. 2a, the severity of systemic inflammation as assessed by the SI score correlated with the number of leukocytes in the blood compartment. Leukocyte counts after severe trauma showed an early peak at the day of admission (day 0), followed by a rapid decline on day 1 to values in the normal range (Fig. 2b). Starting at day 5 after trauma, leukocyte numbers rose again to a second peak on day 11, and then gradually declined during the further course (Fig. 2b). Secondly, we analyzed the changes in leukocyte counts in groups of patients with respect to outcomes. Patients with sepsis showed significantly elevated leukocyte levels, which were particularly pronounced beyond day 4 (Fig. 2c). However, there were no significant differences in the leukocyte course between survivors and nonsurvivors (Fig. 2d).

In contrast to leukocytes, thrombocyte counts did not reflect the severity of inflammation after trauma (Fig. 2e). As for their time course, thrombocyte numbers initially decreased, and beginning on day 4 thrombocyte numbers continuously rose to a plateau on day 13, followed by an undulating course afterwards (Fig. 2f). The levels in the third week after trauma collectively ranged approximately threefold above the upper normal value (Fig. 2f). In patients with sepsis, thrombocyte levels were significantly lower, the increase of thrombocyte numbers was delayed (right shift of the curve), and the plateau in week 3 ranged at significantly lower levels as compared with patients without septic complications (Fig. 2g). A similar pattern was found for mortality, with significantly lower thrombocyte levels in nonsurvivors, suggesting that inadequate increase of thrombocyte numbers after severe trauma is associated with an adverse outcome or with septic complications (Fig. 2h).

### Performance of leukocytes, thrombocytes, and their combination as markers for outcome of trauma patients

**Fig. 3:**
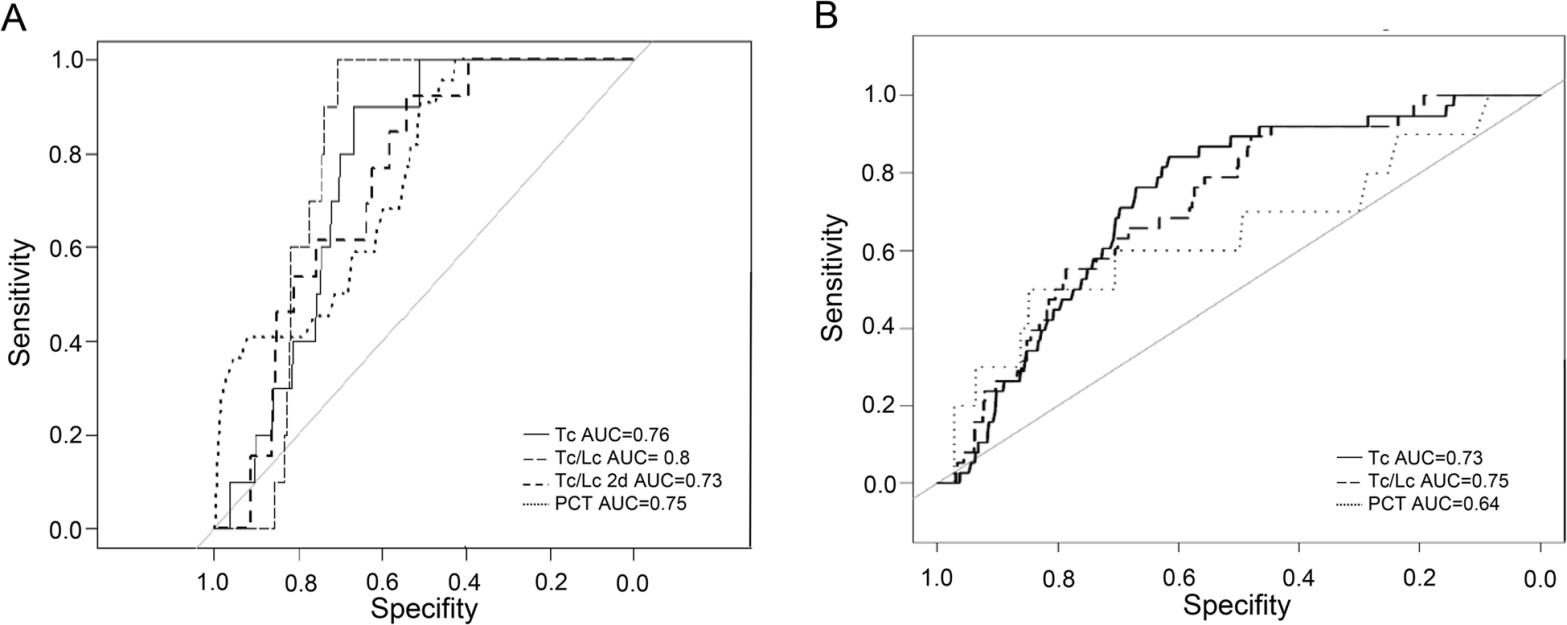
**a** ROC curves for thrombocytes (*Tc*), leukocytes (*Lc*), the combination of the two (*Tc/Lc*), and PCT as “positive control” regarding the outcome (mortality). **b** ROC curve analysis (mortality) for Tc, Tc/Lc, and PCT within 3 days prior to the time point of death. Area under the curve (*AUC*) values are provided

Based on the previous results, prognostic performance of leukocytes and thrombocyte counts and their combination was assessed and compared with the routinely used parameter PCT. Thrombocyte counts showed a performance for mortality in trauma patients with slightly better area under the curve (AUC) values (AUC = 0.76) than PCT (AUC = 0.75), which could be improved when combined with leukocyte counts (AUC thrombocytes/leukocytes = 0.8; Fig. 3a). In relation to the time point of death, thrombocyte/leukocyte levels determined 2 days prior to the event still performed with an AUC value of 0.73 (Fig. 3a). With regard to clinical applicability, the performance of thrombocytes and leukocytes was evaluated within a period of 3 days prior to the time point of death. In this setting, thrombocytes and leukocytes were found to be reliable markers to predict a lethal outcome (AUC thrombocytes = 0.73; AUC thrombocytes/leukocytes = 0.75); with increasing performance, the intervals between sampling and the lethal event were shorter (Fig. 3b).

### Cluster analysis of selected transcriptomic candidates

**Fig. 4:**
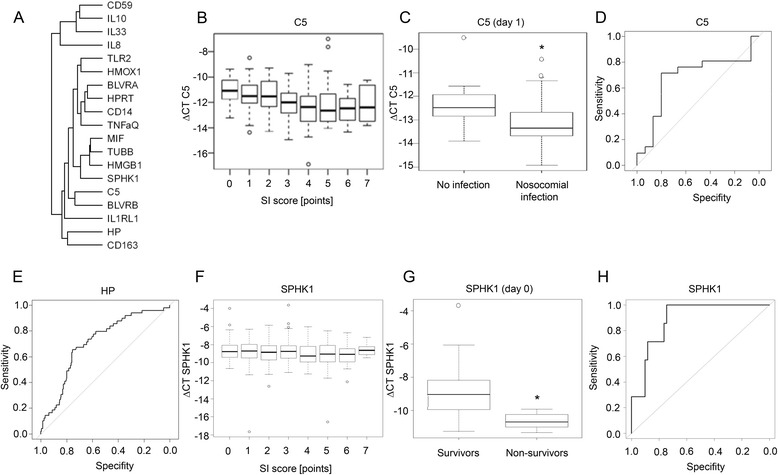
Characterization of selected transcriptomic markers with best performance. **a** Dendrogram depicting unsupervised cluster analysis of all transcriptomic markers: HP, CD163, HMOX1, BLVRA, BLVRB, IL-10, TLR2, CD14, IL-8, HMGB1, IL-33, IL-1RL1, C5, CD59, TNFα, MIF, SPHK1, HPRT, TUBB, and ACTB. **b** Correlation of C5 expression (∆CT) with the systemic inflammation (*SI*) score (*n* ≥ 53 patients). **c** Expression of C5 in leukocytes on day 1 after trauma from patients who developed nosocomial infections during the further course in comparison with patients without infection. ROC curves for **d** C5 (endpoint: nosocomial infection; AUC = 0.68) and **e** HP (endpoint: sepsis; AUC = 0.71). **f** Correlation of SPHK1 expression (∆CT) with the SI score (*n* = 71 patients). **g** Comparison of SPHK1 expression (∆CT) in survivors and nonsurvivors. **h** ROC curve for SPHK1 on day 0 (endpoint: mortality; AUC = 0.89). *p <0.05. *HP* haptoglobin, *SPHK* sphingosine kinase

With the goal of stratifying the importance of the selected genes, an unsupervised clustering was conducted of all selected transcriptomic markers. Expression was adjusted to the housekeeping gene *ACTB* and to baseline levels (day 0; ΔΔCT). As shown in Fig. 4a, HP and CD163 of the heme degradation pathway clustered together, and C5 grouped with BLVRB, while HMOX1 clustered with candidates of the pathogen recognition receptor family (TLR2, CD14) and cytokines (TNFα). Another cluster comprised the DAMP HMGB1, the cytokine MIF, and SPHK1 (Fig. 4a).

### Expression of the top transcriptomic performers C5, HP, and SPHK1 as markers for infectious complications, sepsis, or mortality

We further tested for potential correlations between our candidate genes and clinical outcomes. The expression of C5 (ΔCT) showed only a weak negative correlation with the severity of disease (SI score; Fig. 4b). However, C5 expression on day 1 after trauma was significantly lower in patients who developed nosocomial infections during the further course than those without infectious complications (Fig. 4c), with moderate sensitivity/specificity (AUC = 0.68; Fig. 4d).

In a previous study describing the role of the heme degradation pathway in the development of secondary sepsis in severely injured patients, HP expression in circulating leukocytes reflected the severity of systemic inflammation, and, most importantly, significant upregulation was found in trauma patients with sepsis. In line with these findings, HP was found to be a valid marker for identification of septic complications in trauma patients in the present study (Fig. 4e, AUC = 0.72).

Regarding the expression of SPHK1, no correlation with the SI score was found (Fig. 4f). Strikingly, SPHK1 expression was significantly lower in nonsurvivors than in survivors as early as at the time point of admission to the emergency department (day 0; Fig. 4g). With these profound differences, all nonsurvivors in the cohort of the present study ranged below a threshold of ΔCT ≤ –10 (Fig. 4g). In accord, ROC analysis revealed an outstanding performance for SPHK1 expression on day 0 regarding mortality (Fig. 4h; AUC = 0.89).

### Comparison of performance of individual markers

**Fig. 5:**
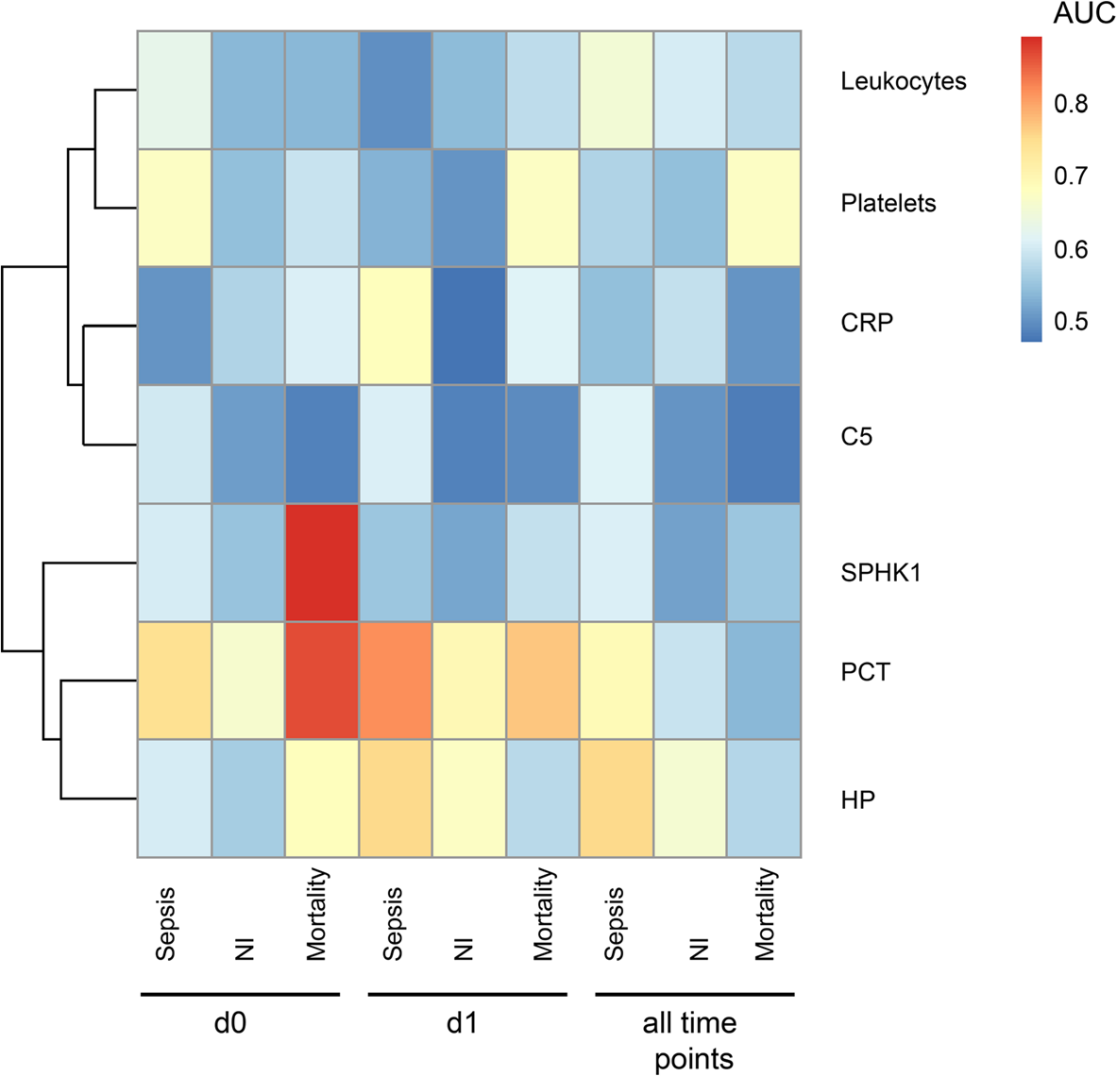
Comparison of the performance (heatmap of AUC values) of selected clinical and transcriptomic parameters regarding specific outcomes (sepsis, nosocomial infections (*NI*), mortality) and time points of assessment (day 0 (*d0*), day 1 (*d1*), all days); *n* = 71 patients. *AUC* area under the curve, *CRP* C-reactive protein, *HP* haptoglobin, *PCT* procalcitonin, *SPHK* sphingosine kinase

Performances of each single marker (leukocytes, thrombocytes, C5, HP, SPHK1, and the routinely used laboratory parameters CRP and PCT) were compared with respect to various outcomes (sepsis, nosocomial infections, mortality) and time points of assessment (day 0, day 1, all days; Fig. 5). As the main results, PCT was found to be a reliable marker for prediction of sepsis and mortality at early time points (day 0, day 1), while HP was the best marker for sepsis when all time points were considered. In accordance with Fig. 4h, SPHK1 showed the best performance among all single markers for mortality on day 0 (Fig. 5).

### Temporal relationship of clinical and transcriptomic candidates

**Fig. 6:**
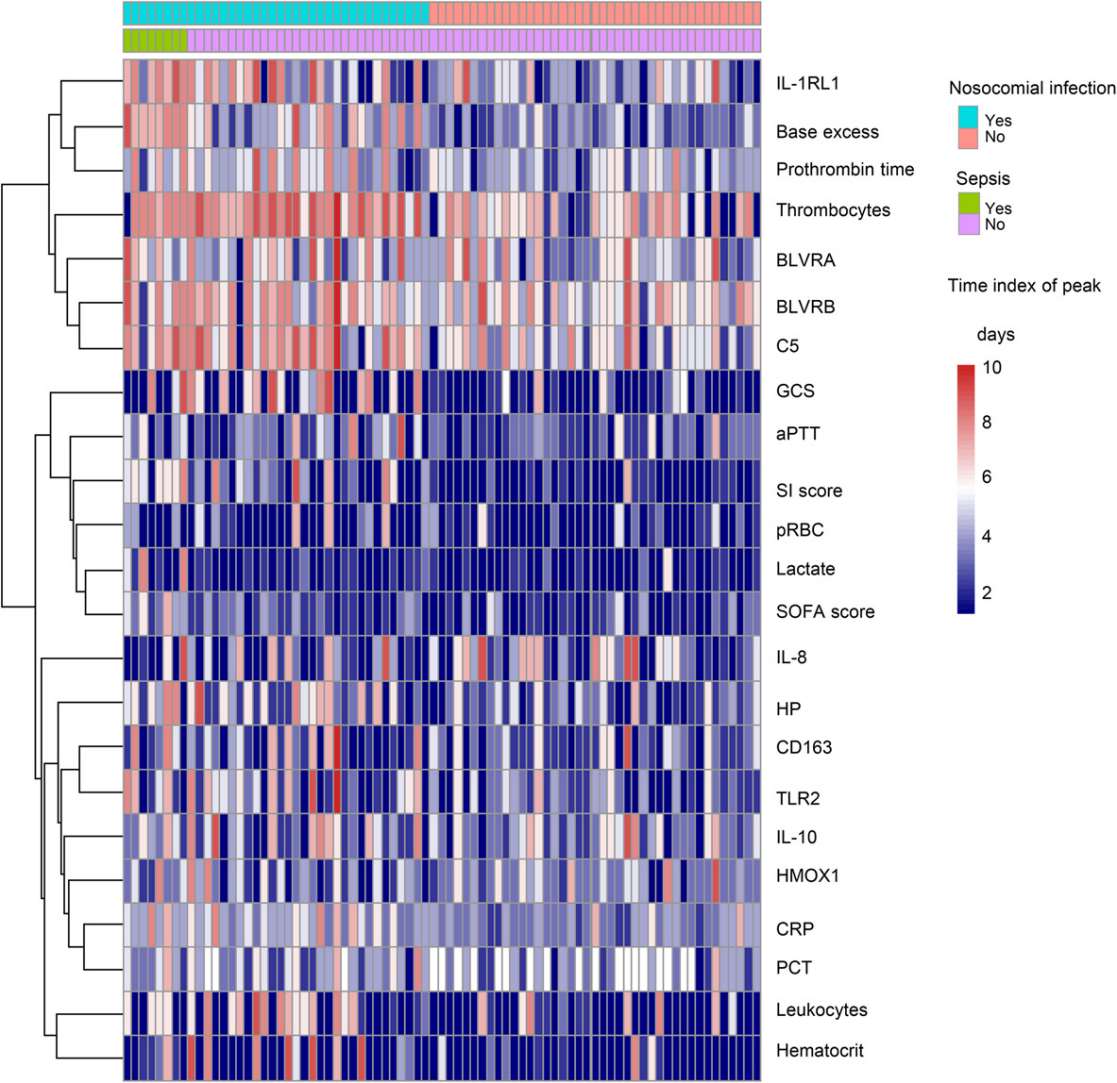
Hierarchical cluster analysis of various clinical and transcriptomic markers with regard to time index of peak measurements (time after injury to reach maximum values) in relation to the binary outcome variables nosocomial infection and sepsis. *n* = 71 patients. *aPTT* activated partial thromboplastin time, *BLVR* Biliverdin reductase, *CRP* C-reactive protein, *HMOX1* heme oxygenase-1, *HP* haptoglobin, *IL* interleukin, IL-*1RL1* interleukin 1 receptor-like 1, *PCT* procalcitonin, *SI score* systemic inflammation score, *SOFA* Sequential Organ Failure Assessment, *TLR* toll-like receptor, *GCS* Glasgow Coma Score, *pRBC* Packed Red Blood Cells

**Fig. 7:**
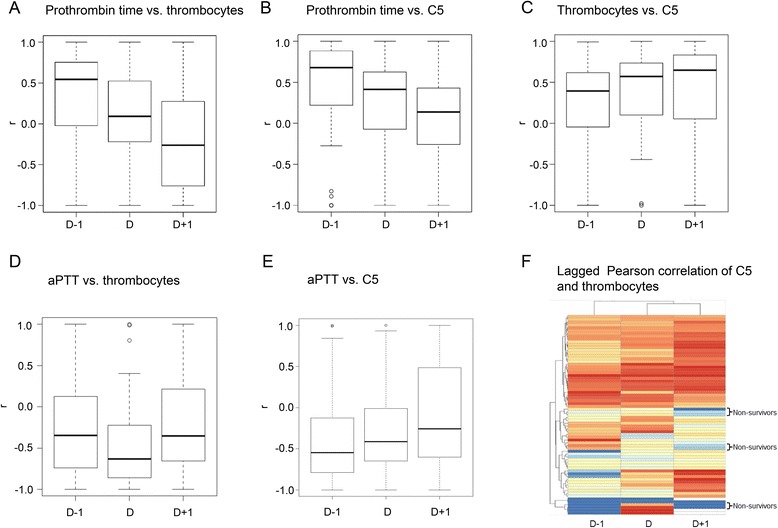
Correlational analyses of C5, thrombocytes, and coagulation tests (prothrombin time; activated partial thromboplastin time (*aPTT*)). Lag effects are reflected by analysis of preceding (*d–1*) and consecutive time points (*d + 1*). Data are presented as box plots of correlation coefficients *r* (*n* ≥53 patients). **a** Prothrombin time vs. thrombocyte counts. **b** Prothrombin time vs. C5 expression (∆CT). **c** Thrombocyte counts vs. C5 expression (∆CT). **d** aPTT vs. thrombocyte counts. **e** aPTT vs. C5 expression (∆CT). **f** Heatmap for lagged Pearson correlation of C5 (∆CT) expression and thrombocyte counts

Hierarchical cluster analysis of various clinical and transcriptomic markers using time index of peak measurements (time after injury to reach maximum values; Fig. 6) was applied in order to identify and illustrate common expression patterns and their temporal relationships in patients with a similar clinical course and outcome with respect to nosocomial infections and sepsis. Patients with infectious complications, including sepsis, showed distinct patterns (Fig. 6, upper left quadrant of heatmap). In this group, C5 clustered with downstream components of the heme degradation pathway (BLVR), thrombocytes, and prothrombin time, suggesting common regulatory mechanisms. The relationship of these peak dynamics is further characterized in Fig. 7 (evaluation of lag effects and trajectories). The remaining transcriptomic candidates of the heme degradation pathway (HP, CD163, HMOX1; IL-10) and IL-8 clustered together, with moderate correlation to nosocomial infections (Fig. 6, lower half of the heatmap). The figure also implies that there are inter-individual differences in the dynamics of all markers, reflecting the heterogeneity of trauma patient cohorts.

### Association and causality of C5, thrombocytes, and prothrombin time

Based on the temporal expression patterns of the cluster analysis presented in Fig. 6, the association between C5 expression, thrombocyte counts, and routine coagulation tests (prothrombin time; Fig. 7a–c), all of which may be affected by or even contribute to trauma-induced coagulopathy, was assessed in further detail. This association was found to underlie lag effects by 1 day (indicated by d–1 or d + 1 in Fig. 7a–c), with changes of the prothrombin time preceding the corresponding alterations of C5 expression or thrombocyte numbers. This association was specific for the prothrombin time but not for the activated partial thromboplastin time (Fig. 7d, e). However, in the setting of the present study, prothrombin alone failed to be a reliable prognostic marker. Instead, lagged correlation analysis of C5 and thrombocytes revealed distinct patterns, with which the nonsurvivors could be discriminated (Fig. 7f). Collectively (Figs. 6 and 7), these analyses reflect the temporal dynamics of the systemic inflammatory response after trauma and provide additional insights as compared with sole correlation analyses. Distinct temporal patterns of certain clinical and transcriptomic features may be used for discrimination of outcomes (e.g., infectious complications and sepsis).

### Decision tree cross-validation

**Fig. 8:**
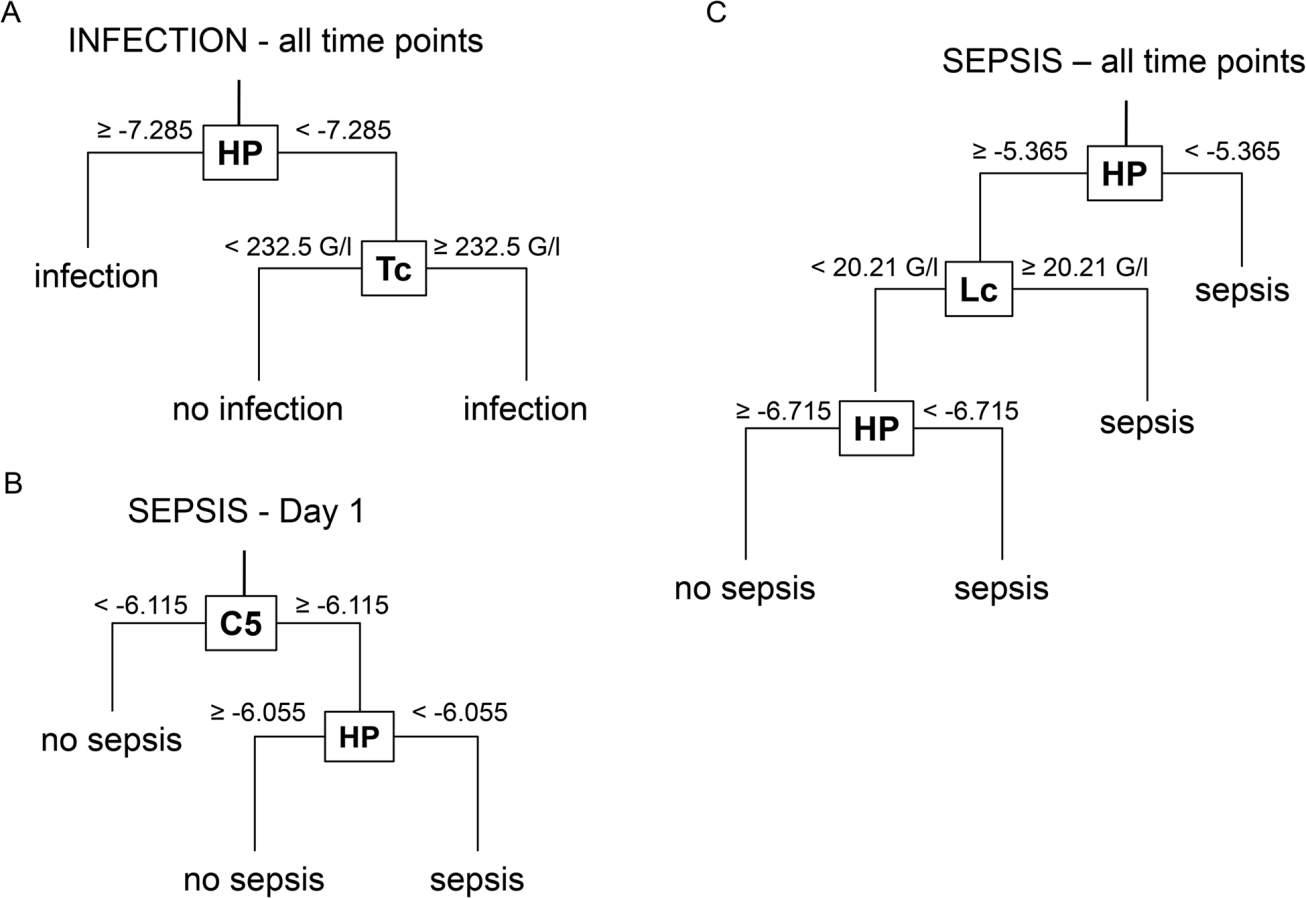
Integrated use of clinical and transcriptomic markers assessed by decision tree cross-validation (10-fold cross-validation; decision trees/candidates were selected upon high specificity). **a** Decision tree for the incidence of nosocomial infections after trauma under consideration of all time points of the observation period (*n* = 413 samples). **b** Assessment of the risk for the development of sepsis during the further course using samples from day 1 after trauma (*n* = 77 samples). **c** Decision tree for sepsis under inclusion of all time points of the observation period (*n* = 502 samples). Threshold levels (ΔCt of gene expression or leukocyte/thrombocyte counts) for the decision of which path is taken are provided in the figures at the corresponding levels. *C5* complement component C5, *HP* haptoglobin, *Lc* leukocytes, *Tc* thrombocytes

Finally, under consideration of all the longitudinal data presented, the combined, hierarchical application of various markers was assessed by decision tree cross-validation. As displayed in Fig. 8, these analyses revealed different combinations of markers depending on the outcome parameter (nosocomial infection; sepsis) and the time point of assessment (day 1 after trauma vs. all time points during the observation period). To evaluate the trauma patients’ risk for developing of nosocomial infections at any time point, the hierarchical combination of HP expression (primary level) and thrombocytes (secondary level) may be used (Fig. 8a; specificity = 0.9097; sensitivity = 0.6154; AUC = 0.7332). Development of sepsis during the further course is indicated by C5 expression on day 1 after trauma followed by assessment of HP expression at the same time point (Fig. 8b; specificity = 0.9565; sensitivity = 0.6250; AUC = 0.7880). When all time points during the observation period are considered, the incidence of sepsis can be assessed by measurement of HP expression at a certain time point (primary level), followed by evaluation of leukocyte counts (secondary level). Here, leukocyte levels greater than 20.21 g/l are indicative for the development of sepsis. For patients with leukocyte levels below this threshold, HP expression can be assessed on the tertiary level for the risk of sepsis (Fig. 8c; specificity = 0.9657; negative predictive value = 0.9174; positive predictive value = 0.6428; AUC = 0.8219).

## Discussion

In the present study, we sought to identify clinical and transcriptomic markers (clinico-transcriptomic analysis) and their combination that correlate with the outcome and indicate the patients’ risk for adverse outcomes and for developing secondary complications following trauma, including nosocomial infections and sepsis. The selection of transcriptomic markers was based on previous findings from whole genome analyses and known mechanisms of the inflammatory response, and comprised various mediators of inflammation (cytokines, complement system), Danger-Associated Molecular Patterns (DAMPs) and Pattern Recognition Receptors (PRRs), and the heme degradation pathway. Clinical markers included standard physiological and laboratory parameters and scoring systems routinely determined in the assessment of trauma patients. In a recent study by our group, the heme degradation pathway has been found to be upregulated in trauma patients who developed sepsis as compared with trauma patients with an uncomplicated recovery. As mentioned in the Introduction, several studies with a similar objective in comparable trauma patient cohorts exist [4, 5, 7, 8]. However, each of these studies, including the present study, revealed a different set of candidate genes to be used as markers in trauma patients, with only little overlap. This discrepancy may be due to differences in the study design, different methods applied (different microarray platforms vs. NanoString vs. PCR), or nonuniform classification of clinical conditions (e.g., complicated discovery vs. sepsis). In the present study, we were able to demonstrate that HP in particular represents a promising marker for the development of sepsis after trauma, which precedes the occurrence of clinical signs of sepsis by at least 1 day. In addition, TLR2 and CD14 were analyzed as representatives of the pattern-recognition receptors which represent another system that was upregulated in sepsis patients of the discovery set as described previously. While the temporal changes and regulation of TLR2 and CD14 could be confirmed in the cohort of the present study, these markers were not found to be superior to clinical parameters and scores with respect to their prognostic performance. Likewise, transcription of the selected cytokines IL-6, TNFα, IL-10, and IL-8 showed differential regulation after trauma but was not found to be expedient to be used as markers for clinical assessment of trauma patients, in contrast to their protein equivalents. Although playing a central role in the initiation of the inflammatory response, the expression of DAMPs, with HMGB1 in particular, showed only small changes after trauma. In fact, HMGB1 expression in leukocytes was similar to the housekeeping genes *ACTB* or *TUBB*. These findings are in striking contrast to the pattern of the circulating HMGB1 protein in trauma patients, with an early peak immediately after trauma [17, 18]. This discrepancy between proteomic and transcriptomic expression patterns suggests either that preformed HMGB1 is released upon trauma or that it is predominantly released from cell types other than circulating leukocytes.

Among the candidates included in the analyses by “knowledge-based selection”, C5 and SHPK1 expression appeared to be proper markers to assess the patients’ risk for adverse outcomes (infectious complications, mortality) in the early phase (day 0, day 1) after trauma, in accordance with their central roles in the pathophysiology in systemic inflammation.

Among all clinical parameters, leukocytes and thrombocytes were found to be the candidates with reasonable performance. As was to be expected, leukocytes were increased after trauma peaking at the end of the second week. Leukocyte counts reflected the severity of systemic inflammation and were significantly elevated in patients who developed secondary sepsis, but there were no differences between survivors and nonsurvivors. As for the thrombocyte profile in trauma patients, significant differences were found in patients who developed sepsis or who did not survive. In both subgroups, a right shift of the curve occurred, and the typical plateau in the third week after trauma was reached at significantly lower levels. This “relative thrombocytopenia” was also associated with multiorgan dysfunction (SOFA score >8 points). Thrombocytes thus represent an all-round marker for a “complicated recovery” and adverse outcome, whose discrimination capability and performance may even be improved when combined with other markers. In this context, it is important to note that not the absolute values of thrombocyte counts, but the delayed increase during the first week (right shift) and the lower level of the plateau during the third week in relation to patients with an uncomplicated recovery were indicative for adverse events. It is well established that following initial thrombocytopenia due to consumption, thrombocyte counts increase after trauma in response to release of thrombopoietin [36]. In accord with our findings, it has been suggested that increased thrombocyte levels after trauma are associated with an improved survival, while the significance of trauma-associated thrombocytosis remained unclear [37]. Our findings with detailed analysis of the kinetics of circulating thrombocytes by daily measurements over a period of 3 weeks suggest that late thrombocytosis may be required for proper post-injury regeneration and tissue repair, whereas the early decline may be due to consumption and trauma-associated coagulopathy. Regarding their functional role, it became evident that thrombocytes are closely linked to immunity [38]: thrombocytes are activated by DAMPs and Pathogen-Associated Molecular Patterns (PAMPs) and express immune receptors on their surface, including complement receptors and PRRs (TLR) [39, 40]. Inappropriate activation of thrombocytes during systemic inflammation is a major contributor to disseminated intravascular coagulation, which, in turn, causes early thrombocyte consumption that is related to mortality [41].

In this translational study, application of integrated clinico-transcriptomic analyses was found to be an effective approach for risk stratification and outcome prediction in polytrauma patients, which is in line with several recent reports [5, 42, 43]. By systematic statistical dissection of data from large-scale analyses, we were able to identify a set of markers with reliable prognostic performance.

Our data suggest that C5 expression might be used as an early marker (day 1 after trauma) for identification of trauma patients at risk for the development of nosocomial infections/sepsis. HP was found to be a reliable marker for the development of secondary sepsis, which is based on upregulation of the heme degradation pathway by free heme after allogenic blood transfusion or release of myoglobin from traumatic tissue damage, as implied by a most recent study. As another promising candidate, SPHK1 has been found to be an early marker for prediction of mortality, with an excellent performance as early as at the day of admission (AUC = 0.89). Functionally, SPHK signaling is known to play a crucial role in the development, differentiation, activation, and proliferation of immune cells [44, 45], and its product sphingosine-1 phosphate has been shown to attenuate multiple organ dysfunction in an experimental model of trauma/hemorrhagic shock [46]. In comparison with the performance of each single marker alone (Fig. 5), these data imply that in combination the sensitivity and specificity of the markers can be improved. Another advantage for inclusion of transcriptomic markers in the assessment of trauma patients is that changes in the transcription occur earlier than changes of clinical parameters and scores, and precede the clinical event (e.g., sepsis). In the case of C5 and SPHK1, differences in patients were even evident as early as on day 0 (SHPK1) and day 1 (C5) after trauma, allowing early and timely identification of patients at risk for infectious complications and an adverse outcome.

From a pathophysiological point of view, the markers identified by the present study are not only complementary to each other regarding their prognostic performance but are also functionally related. A previous study [47] demonstrated that systemic complement activation already occurs minutes after severe trauma. Due to its close interaction with the coagulation cascade [48, 49], complement activation may thereby contribute to traumatic coagulopathy. This is reflected by impaired prothrombin times, which have been described previously to predict an unfavorable outcome [50]. Trauma-induced coagulopathy is also hallmarked by early thrombocyte dysfunction. In our study, thrombocyte counts and C5 expression were linked through the prothrombin time, and lagged correlations of both markers showed a distinct pattern in those trauma patients who did not survive. Thrombocyte-derived microvesicles trigger the upregulation of SPHK1 in monocytic cells in inflammation and sepsis [51]. Moreover, SPHK-1 can be activated by and regulates signaling through C5a receptors [52, 53], which also play central roles in the initiation and progression of inflammation in sepsis [22].

In summary, our findings indicate that integration of clinical and transcriptomic markers allows risk stratification and prediction of infectious complications and an adverse outcome in trauma patients. In the cohort of the present study, leukocytes, thrombocytes, and the expression of SPHK1, C5, and HP in leukocytes have been identified as markers with the best performance which might be used for assessment of trauma patients.

A hypothetical algorithm of how the information from the present study might be transferred to the clinical setting is as follows: on the day of trauma (day 0), patients with a high risk of mortality could be identified by SPHK1 expression, and these patients may be monitored by combined assessment of C5 expression and thrombocyte count during the further course. The expression of C5 1 day after trauma (day 1) may indicate the patients’ risk for nosocomial infections and sepsis. In this subgroup, the risk to develop secondary sepsis could further be assessed by HP expression. In combination with HP expression, leukocyte levels may help stratify the patients’ risk for development of sepsis at any time point during the course after trauma.

## Conclusions

The integrated application of clinical and transcriptomic markers (clinico-transcriptomic analyses) improves the prognostic performance in trauma patients and may represent a useful tool for individual risk profiling and stratification. The clinical practicability of this approach needs to be validated in future prospective studies in independent trauma patient cohorts.

## Key messages

- Expression changes of C5, HP, and SPHK1 in whole blood from trauma patients have been identified as markers for infectious complications, sepsis, or mortality, respectively.
- Leukocyte counts after trauma reflect the severity of systemic inflammation and correlate with the development of sepsis, while thrombocyte counts are associated with adverse outcomes in severely injured patients.
- The integrated use of clinical and transcriptomic markers improves the prognostic performance and may represent a useful tool for individual risk stratification in trauma patients.

## Additional files

Additional file 1: Table S1. is presenting the SI score. (DOCX 16 kb) Additional file 2: Table S2. is presenting the primer sequences. (DOCX 16 kb) Additional file 3: Table S3. is presenting the timing of endpoints. (DOC 28 kb)

## Abbreviations

ANOVA: Analysis of variance
AUC: Area under the curve
BLVR: Biliverdin reductase
CRP: C-reactive protein
CT: Cycle threshold
GO: Gene Ontology
GSEA: Gene set enrichment analysis
HMGB1: High mobility group box protein-1
HMOX1: Heme oxygenase-1
HP: Haptoglobin
IL: Interleukin
IL-1RL1: Interleukin 1 receptor-like 1
ISS: Injury Severity Score
MIF: Migration inhibitory factor
MOF: Multiorgan failure
PCT: Procalcitonin
ROC: Receiver operating characteristic
RQI: RNA quality indicator number
SI score: Systemic inflammation score
SOFA: Sequential Organ Failure Assessment
SPHK: Sphingosine kinase
TLR: Toll-like receptor
TNFα: Tumor necrosis factor alpha
