# Supplementary material for: Improvement of prognostic performance in severely injured patients by integrated clinico-transcriptomics: a translational approach
Source: Crit Care. 2015 Nov 26;19:414. doi: 10.1186/s13054-015-1127-y (PMC4660831; doi:10.1186/s13054-015-1127-y)
Supplement: Additional file 1: Table S1. — is presenting the SI score. (DOCX 16 kb) [file 13054_2015_1127_MOESM1_ESM.docx]

| **Additional file 1: Table S1.** Systemic inflammation (SI) score [32]. |  |
| --- | --- |
|  |  |
|  |  |
| **Systemic inflammation criteria** | **Points** |
|  |  |
| Body temperature ≥ 38°C or ≤ 36°C | 1 |
| Tachykardia ≥ 90 bpm | 1 |
| Tachypnea ≥ 20/min or hyperventilation (PaCO_2_ ≤ 4,3 kPa) | 1 |
| Leukocytosis (≥ 12 G/l) or leukopenia (≤ G/l) | 1 |
| Thrombocytopenia within 24 h (drop of 30% in platelet count from patient's high in past 3d or platelet count < 100.000/mm^3^) | 1 |
| Arterial hypotension (systolic blood pressure ≤ 90 mmHg or MAP ≤ 70 mmHg) | 1 |
| Hypoxia (PaO_2_ ≤ 10 kPa) or PaO_2_/FiO_2_ ratio ≤ 33 kPa | 1 |
| Infection | 1 |
| **TOTAL** | **8** |
