# Supplementary material for: Improvement of prognostic performance in severely injured patients by integrated clinico-transcriptomics: a translational approach
Source: Crit Care. 2015 Nov 26;19:414. doi: 10.1186/s13054-015-1127-y (PMC4660831; doi:10.1186/s13054-015-1127-y)
Supplement: Additional file 2: Table S2. — is presenting the primer sequences. (DOCX 16 kb) [file 13054_2015_1127_MOESM2_ESM.docx]

| **Additional file 2: Table S2.** Primer sequences. | |  |  |  |  |
| --- | --- | --- | --- | --- | --- |
|  |  |  |  |  |  |
|  |  |  |  |  |  |
| **Target** | **Forward 5‘-3‘** | **Reverse 5‘-3‘** | **Product (bp)** | **Annealing/ Extension** | **Efficiency** |
| IL8 | CAGGAATTGAATGGGTTTGC | AGCAGACTAGGGTTGCCAGA | 139 | 62 | 2,00 |
| IL10 | ACTTTAAGGGTTACCTGGGTTGC | TCACATGCGCCTTGATGTCTG | 111 | 60 | 2,00 |
| IL33 | ACCAGAGCCTAGATGAGACACCG | GCCAGGGTCAGAAGGGATGGT | 93 | 63 | 0,98 |
| CD14 | ATGCCTCCGCTGCCTCTGGA | GCGAGCCAAGAACGCCCTGT | 92 | 61 | 2,00 |
| CD59 | AGGCAAACAGCACCCCAGGT | GCCACAGCCCTCTCCAGCCA | 54 | 63 | 2,00 |
| CD163 | GCAAGACCCCGTGTGAGGGC | GATCCCCAGGCACCAAGCGT | 55 | 64 | 2,00 |
| TLR2 | GATGCCTACTGGGTGGAGAA | CCACTTGCCAGGAATGAAGT | 102 | 61 | 2,00 |
| BLVRA | TGCGGATGAGGGACTTGCGG | GGCGACCTCCACCTCTTGGC | 149 | 62 | 2,00 |
| BLVRB | AGGCGGTGCAAGCAGGTTACG | GCCCAGCCACGGTCTTGTCC | 138 | 62 | 2,00 |
| IL1RL1 | CCAGTAATCGGAGCCCCTGCAC | TAAGCTGCCACAGGACGGCA | 127 | 62 | 1,98 |
| HMGB1 | CGTCTGGCTCCCGCTCTCAC | CGAGGCACAGAGTCGCCCAG | 103 | 63 | 1,96 |
| C5 | TGTCAAGGCAAAGGTGTTCA | CCCTCCACAGCAGACATTTT | 150 | 61 | 2,00 |
| HMOX1 | CCAGCAACAAAGTGCAAGATT | GTGTAAGGACCCATCGGAGA | 102 | 62 | 1,95 |
| HP | TGCTGCCTGTGGCTGACCAA | CGGGGACTGTGCTGCCTTCA | 60 | 61 | 1,99 |
| SPHK1 | TGGACAGTGGTGCCCGACGA | CCCGCCCGCACGTAGAACAG | 134 | 62 | 2,00 |
| MIF | GCGGGTCTCCTGGTCCTTCT | GCGGGGCACGTTGGTGTTTA | 62 | 64 | 2,00 |
| HPRT1 | CCTGGCGTCGTGATTAGTGAT | AGACGTTCAGTCCTGTCCATAA | 131 | 61 | 2,00 |
| ACTB | GGCATGGGTCAGAAGGATT | AGGTGTGGTGCCAGATTTTC | 133 | 61 | 2,00 |
| TUBB | TTGCCCCTCTCACCAGCCGT | CGGAAGACAGCAGCCACGGT | 145 | 62 | 2,00 |
|  |  |  |  |  |  |
