# Supplementary material for: Improvement of prognostic performance in severely injured patients by integrated clinico-transcriptomics: a translational approach
Source: Crit Care. 2015 Nov 26;19:414. doi: 10.1186/s13054-015-1127-y (PMC4660831; doi:10.1186/s13054-015-1127-y)
Supplement: Additional file 3: Table S3. — is presenting the timing of endpoints. (DOC 28 kb) [file 13054_2015_1127_MOESM3_ESM.doc]

**Additional file 3: Table S3**.
